# Distinct effects of ASD and ADHD symptoms on reward anticipation in participants with ADHD, their unaffected siblings and healthy controls: a cross-sectional study

**DOI:** 10.1186/s13229-015-0043-y

**Published:** 2015-08-28

**Authors:** Eelco V. van Dongen, Daniel von Rhein, Laurence O’Dwyer, Barbara Franke, Catharina A. Hartman, Dirk J. Heslenfeld, Pieter J. Hoekstra, Jaap Oosterlaan, Nanda Rommelse, Jan Buitelaar

**Affiliations:** Department of Cognitive Neuroscience, Donders Institute for Brain, Cognition and Behaviour, Radboud University Medical Center, PO Box 9101, 6500 HB Nijmegen, The Netherlands; Centre for Cognitive Neuroimaging, Donders Institute for Brain, Cognition and Behaviour, Radboud University, PO Box 9101, 6500 HB Nijmegen, The Netherlands; Department of Human Genetics, Donders Institute for Brain, Cognition and Behaviour, Radboud University Medical Center, PO Box 9101, 6500 HB Nijmegen, The Netherlands; Department of Psychiatry, Donders Institute for Brain, Cognition and Behaviour, Radboud University Medical Center, PO Box 9101, 6500 HB Nijmegen, The Netherlands; Department of Psychiatry, University Medical Centre Groningen, University of Groningen, PO Box 30.001, 9700 RB Groningen, The Netherlands; Department of Clinical Neuropsychology, VU University, Van der Boechorststraat 1, 1081 BT Amsterdam, The Netherlands; Karakter, Child and Adolescent Psychiatry University Center Nijmegen, Reinier Postlaan 12, 6525 GC Nijmegen, The Netherlands

**Keywords:** ADHD, ASD, Reward, Reward anticipation, Comorbidity

## Abstract

**Background:**

Autism spectrum disorder (ASD) traits are continuously distributed throughout the population, and ASD symptoms are also frequently observed in patients with attention-deficit/hyperactivity disorder (ADHD). Both ASD and ADHD have been linked to alterations in reward-related neural processing. However, whether both symptom domains interact and/or have distinct effects on reward processing in healthy and ADHD populations is currently unknown.

**Methods:**

We examined how variance in ASD and ADHD symptoms in individuals with ADHD and healthy participants was related to the behavioural and neural response to reward during a monetary incentive delay (MID) task. Participants (mean age: 17.7 years, range: 10–28 years) from the NeuroIMAGE study with a confirmed diagnosis of ADHD (*n* = 136), their unaffected siblings (*n* = 83), as well as healthy controls (*n* = 105) performed an MID task in a magnetic resonance imaging (MRI) scanner. ASD and ADHD symptom scores were used as predictors of the neural response to reward anticipation and reward receipt. Behavioural responses were modeled using linear mixed models; neural responses were analysed using FMRIB’s Software Library (FSL) proprietary mixed effects analysis (FLAMEO).

**Results:**

ASD and ADHD symptoms were associated with alterations in BOLD activity during reward anticipation, but not reward receipt. Specifically, ASD scores were related to increased insular activity during reward anticipation across the sample. No interaction was found between this effect and the presence of ADHD, suggesting that ASD symptoms had no differential effect in ADHD and healthy populations. ADHD symptom scores were associated with reduced dorsolateral prefrontal activity during reward anticipation. No interactions were found between the effects of ASD and ADHD symptoms on reward processing.

**Conclusions:**

Variance in ASD and ADHD symptoms separately influence neural processing during reward anticipation in both individuals with (an increased risk of) ADHD and healthy participants. Our findings therefore suggest that both symptom domains affect reward processing through distinct mechanisms, underscoring the importance of multidimensional and multimodal assessment in psychiatry.

**Electronic supplementary material:**

The online version of this article (doi:10.1186/s13229-015-0043-y) contains supplementary material, which is available to authorized users.

## Background

Autism spectrum disorder (ASD) is a prevalent neurodevelopmental disorder characterized by social, communicative and behavioural deficits [1]. ASD traits are continuously distributed in the general population, with symptomatology below the clinical threshold for diagnosis being relatively common [2–4]. Research into the broader ASD-related phenotype is especially relevant since ASD symptoms are elevated in various clinical populations, particularly in patients with attention-deficit/hyperactivity disorder (ADHD).

ADHD, a neurodevelopmental disorder characterized by inattentiveness and/or hyperactivity and impulsivity, has been associated with high ASD comorbidity and elevated levels of ASD symptoms compared to the general population [5–7]. The high levels of comorbidity of ADHD and ASD could be due to a shared aetiology, and studies have indeed shown psychopathological, neuropsychological, neuroimaging and genetic overlap between the disorders [8–10]. How the two symptom domains interact in their effects on cognition, however, remains largely unknown.

One area where ASD and ADHD effects could interact is during the processing of reward. Both disorders have been linked to abnormalities in the frontal-striatal neural circuits associated with reward processing; whether this is the result of similar pathophysiological mechanisms is unclear [7, 11]. Summarizing the literature, it appears that ASD is related to abnormalities in the processing of certain types of reward rather than associated with a general reward-processing deficit [11, 12]. The processing of social and monetary reward has generally been associated with diminished activity in fronto-striatal areas in ASD versus control participants [13–17]; in contrast, some studies have also reported ASD-related hyperactivity during monetary reward processing in brain regions outside the traditional reward circuit [18, 16]. Increased responses to other types of reward (food cues, faces and images of personal relevance) have also been observed in participants with ASD in the insula [19, 18], amygdala [18] and (pre)frontal cortex [16, 18]. Reduced motivation to obtain social and monetary rewards [20] combined with increased motivation to pursue personally relevant stimuli could explain these bidirectional findings in ASD [16, 19].

In ADHD, both hypoactivity and hyperactivity in reward circuits in response to reward have been reported. The current consensus is that ADHD is characterized by decreased striatal activation during reward anticipation [21], but increased prefrontal and striatal responses during reward receipt, compared to typically developing controls [22–25].

Only a few neuroimaging studies on reward processing to date have included participants with ASD and ADHD [11, 26, 27]. In a study by Kohls and co-workers, participants with ADHD displayed increased striatal and prefrontal activation during receipt of monetary reward (compared to control and ASD groups), whereas the presence of ASD was associated with striatal hypoactivity for both monetary and social reward conditions, in line with previous research in ADHD and ASD samples [11]. Chantiluke and colleagues compared the association between behavioural and neural responses related to temporal discounting in ADHD, ASD, comorbid ADHD/ASD and healthy controls. Besides shared abnormalities in all patient groups, they also found ASD-specific differences in the insula, and cerebellar deviations partially shared between ADHD and comorbid participants [27]. A pharmacological study from the same lab showed that fluoxetine (a selective serotonin reuptake inhibitor) had different effects on the neural signatures of reward reversal in ASD and ADHD [26]. ASD-related hypoactivation of medial prefrontal cortex (mPFC) under placebo was normalized under fluoxetine conditions, whereas participants with ADHD displayed mPFC activation similar to controls under placebo but hypoactivation after taking fluoxetine. In addition, both ASD and ADHD showed hypoactivation of the precuneus during reward reversal under placebo conditions compared to controls, suggesting that reward reversal is associated with both common and dissociative neural abnormalities in ADHD and ASD [26].

The previous studies therefore suggest that ASD and ADHD can have both shared and distinct effects on reward processing. In addition, some evidence suggests that the cognitive dysfunctions of comorbid ASD and ADHD are not simply a combination of those of ADHD and ASD, but can be qualitatively different and/or more severe [8, 27, 28]. However, much is still unknown about the specific and combined effects of ASD and ADHD symptoms on reward processes within the same study population.

Hence, in the current study, we investigated these effects in a large well-described sample that included individuals with ADHD, their unaffected siblings and healthy control participants. Unaffected siblings were included as they are known to present with increased ASD symptom levels compared to healthy controls [29]. Reward processing was measured using functional magnetic resonance imaging (fMRI) during a monetary incentive delay (MID) task, a commonly used reward task that reliably elicits activity in reward circuits [30]. By measuring both ADHD and ASD dimensionally, we could systematically investigate the separate and cumulative impact of both factors. With this approach, we aimed to gain insight into both the effects of comorbid ASD and ADHD symptoms in individuals with ADHD and to improve our understanding of the impact of ASD and ADHD traits in unaffected populations.

We expected that higher levels of ASD symptoms would be associated with activity changes in fronto-striatal regions and the insula during reward anticipation and receipt based on previous studies [12–19, 26, 27]. In contrast, ADHD symptoms were expected to relate to decreased striatal activation during reward anticipation, and increased fronto-striatal activation during reward receipt [11, 21].

Based on our analyses, we can report two main findings in this article. First, we observed that participants with more ASD symptoms showed increased activity in the insula during reward anticipation. Second, we found that higher ADHD symptom levels were associated with decreased activity in the dorsolateral prefrontal cortex during reward anticipation. We found no effects during reward receipt or any interaction between the ADHD and ASD effects.

## Methods

### Setup

The current investigation was conducted as part of the Dutch multisite NeuroIMAGE project ([31], http://www.neuroimage.nl/). The NeuroIMAGE study was approved by the local ethics committee (CMO Regio Arnhem – Nijmegen; 2008/163; ABR: NL23894.091.08). For details on recruitment of participants and a description of all study procedures in NeuroIMAGE, see [31]. Critically, this cross-sectional study uses a subsample of the dataset used in von Rhein and colleagues [32], in which reward processing was compared between healthy participants and participants with a clinical diagnosis of ADHD (Additional file 1).

### Monetary incentive delay task

Participants performed a monetary incentive delay (MID) task while undergoing MRI [33]. Participants were instructed to respond as quickly as possible to a target (a white circle) by pressing a button. Responses were correct when given within 270–500 ms after target onset; specifically, the response window was adapted to approximate a 33 % hit rate. Although MID tasks typically use higher reward probabilities, the current task design has been used successfully in the past and has been shown to reliably engage the fronto-striatal reward circuit [34–36]. Each correct response (“hit”) shortened the window by 20 ms while each incorrect response (“miss”) increased it by 10 ms. Response windows were adapted for reward and non-reward conditions separately to equalize the amount of hits on both trial types. Although this method minimized differences in hit rates between conditions, it did so at the cost of losing hit rate as a useful index of behavioural performance. Behavioural outcome was therefore assessed using the reaction times in the reward and non-reward condition. Targets were preceded by a cue (a filled square, duration: 3.5–8.5 s) with variable colour coding (green for reward, red for no reward) that deterministically predicted whether a reward could or could not be obtained on the current trial. Reward consisted of 0.20 € per correct response in the reward condition. The outcome of each trial was displayed for 1650 ms after response. Trials were concluded by a fixed inter-trial interval (the presentation of a blank screen) of 500 ms. The timing between events was not jittered. In total, the task consisted of 25 reward and 25 non-reward trials, supplemented by 25 null events. Null events were trials with only a fixation cross and required no response. Participants were given standardized instructions and performed a block of practice trials before starting the task. Trial order was pseudo-randomized, and the total duration of the experiment was 12 min. The experiment concluded by showing the total amount of money awarded on the screen; this reward was subsequently transferred to the participant’s bank account.

### Participant selection

MRI data for the MID task was available for 564 participants from NeuroIMAGE, which represented all NeuroIMAGE participants who had no MRI contraindications and were willing to undergo MR scanning [31]. Here, participants were included in the analyses if they either 1) had a diagnosis of ADHD, 2) were unaffected siblings of participants diagnosed with ADHD or 3) were unrelated and unaffected control participants. Healthy participants with siblings with ADHD were not included in the control group. Participants suffering from acute psychiatric conditions other than ADHD were excluded. Furthermore, participants were excluded if technical problems occurred during MRI or any scientifically or clinically relevant incidental findings were observed. Additionally, participants who displayed excessive movement (3 movements of 4 mm or more) during MRI were excluded to safeguard data quality. Only 1 participant was included with more than 0 but less than 3 large movements; this participant showed 1 such shift during MRI acquisition. Participants excluded for excessive motion were generally younger, had a higher chance of being diagnosed with ADHD and generally displayed higher Conners Hyperactivity scores than included participants (Additional file 2). Although removal of these participants from our analyses could result in a slight bias, it is a practical reality that such a subpopulation is not optimally suited for MRI studies, and acquiring data of a quality sufficient for analysis was considered more important. Behaviourally, any participant with <5 correct responses in the reward or non-reward condition was excluded to improve our statistical power to detect differences. Participants excluded for this reason tended to be younger; participants with ADHD were overrepresented in the excluded sample but excluded participants with ADHD did not differ in ADHD or ASD symptom scores (or other demographics besides age) compared to included participants with ADHD (Additional file 2). The small bias in the sample used for our analyses that resulted from this exclusion procedure was again preferred over including participants for whom the reward-related neural processes could not be estimated satisfactorily. Finally, only participants were included for whom complete CSBQ questionnaire data were available. A complete exclusion flowchart can be found in Additional file 1.

After this exclusion process, 136 participants with ADHD (“ADHD”), 83 siblings (“SIBS”) and 105 healthy controls (“CON”, total *N* = 324) were available for analysis (for demographics, see Table 1). Participants with ADHD were confirmed to have a clinical diagnosis of ADHD (Additional file 3). A subset of the ADHD group also had comorbid diagnoses of oppositional defiant disorder or conduct disorder (ODD/CD) (*n* = 39). Participants had no other diagnosis of any neurological disorder or learning disability, were 10–28 years of age, had an IQ ≥70, had no MRI contraindications, were confirmed to be off-medication at the time of testing for at least 48 h and were of European-Caucasian descent. Written informed consent was obtained from all participants.

**Table 1:** Participants in the current study

	ADHD	Siblings	Control	
	*N* = 136	*N* = 83	*N* = 105	
	Mean ± SD	Mean ± SD	Mean ± SD	Comparison*
Age (years)	17.71 ± 3.04	18.36 ± 3.78	17.18 ± 3.01	ADHD = SIBS + CON; SIBS > CON
IQ	98.40 ± 14.80	99.11 ± 14.00	107.77 ± 13.91	CON > (ADHD = SIBS)
Conners T Score (combined scales)	69.76 ± 12.92	47.73 ± 6.69	45.38 ± 4.52	ADHD > SIBS > CON
CSBQ ASD	10.56 ± 9.44	9.14 ± 10.10	3.43 ± 4.54	(ADHD = SIBS) > CON
CSBQ Lack of Social Interest	3.89 ± 4.35	3.27 ± 4.14	1.12 ± 2.12	(ADHD = SIBS) > CON
CSBQ Problems with Social Understanding	3.93 ± 3.80	3.36 ± 3.75	1.38 ± 2.12	(ADHD = SIBS) > CON
CSBQ Stereotypical Behaviour	1.43 ± 2.32	1.42 ± 2.23	0.45 ± 1.06	(ADHD = SIBS) > CON
CSBQ Resistance to Change	1.32 ± 1.65	1.10 ± 1.67	0.48 ± 1.01	(ADHD = SIBS) > CON
Reward Hit rate	.361 ± 0.06	.360 ± 0.06	.362 ± 0.06	ADHD = SIBS = CON
No Reward Hit rate	.335 ± 0.07	.333 ± 0.06	.335 ± 0.06	ADHD = SIBS = CON
RT Reward Hits (ms)	292 ± 38.02	284 ± 67.64	283 ± 66.45	ADHD = SIBS = CON
RT No Reward Hits (ms)	326 ± 54.12_	314 ± 79.54	310 ± 75.86	ADHD = SIBS = CON
RT Reward Miss (ms)	294 ± 40.10	283 ± 64.40	283 ± 64.40	ADHD = SIBS = CON
RT No Reward Miss (ms)	331 ± 59.06	311 ± 73.36	309 ± 78.13	ADHD > CON, ADHD = SIBS, SIBS = CON
Adult^a^	49.3 %	44.6 %	39.0 %	Equal**
Site^b^	40 %	40 %	59 %	Unequal**
Sex	69 % M	45 % M	45 % M	Unequal**

*SD* standard deviation, *ADHD* participants with ADHD, *SIBS* unaffected siblings, *CON* unrelated control participants, *M* male.

*Comparisons were made using independent sample *t* tests at *p* < 0.05; **equality of the distributions across participant groups was tested using Pearson’s Chi Square Tests at *p* < 0.05

^a^Percentage of participants aged 18 years or older

^b^Percentage of participants scanned in Amsterdam (the remainder was scanned in Nijmegen)

### Children’s Social Behavior Questionnaire

ASD symptoms were measured with the Children’s Social Behavior Questionnaire (CSBQ; [37]). The CSBQ was developed to measure the whole spectrum of ASD, including milder, subclinical symptoms, and includes items that directly refer to the DSM-5 criteria for ASD as well as items that measure additional symptoms associated with ASD [38]. It consists of 49 items divided into 6 subscales. The six subscales are (1) “Not Tuned” (deficits in tuning emotions and behaviour to the current situation), (2) “Lack of Social Interest” (reduced social interest, motivation and reciprocity), (3) “Orientation Problems”, (problems with orientation in space and time), (4) “Not Understanding”, (problems with understanding social context), (5) “Stereotypic Behaviour”, (repetitive motor and sensory behaviour and stereotypy) and (6) “Resistance to Change” (fear and resistance to change). CSBQ items from subscales 2, 4, 5 and 6 refer directly to the clinical criteria for ASD from DSM-5; subscales 1 and 3 instead index other impairments typically associated with ASD but not specific to this disorder (e.g. executive functioning deficits and social-disruptive behaviour) [39]. Items are scored by parents or legal guardians on a three-point scale ranging from “does not apply” via “occasionally applies” to “clearly or often applies”. Subscale scores are calculated by summing up the scores of all contributing items. In this study, a composite score was used of the four CSBQ subscales that target deficits specific to ASD (CSBQASD, the sum of scores on scales 2, 4, 5 and 6) to isolate the contribution of ASD symptoms from those of other disorders.

### Conners Parent Rating Scale

As an analog to the dimensional CSBQASD score, we used the Conners Parents Rating Scale Revised-Long Version (CPRS-R-L) [40, 41] as a dimensional index of ADHD severity. Specifically, we used the combined raw score of the ratings on the DSM inattentive and DSM hyperactive/impulsive subscales as our measure of ADHD symptoms. In addition, we investigated the individual impact of the subscales by including their raw scores as separate regressors in supplementary analyses (Additional file 4).

### Medication status

Although all participants with ADHD were off medication for at least 48 h before our measurements were taken, their history of medication use was not equal. Permission was sought from each participant to obtain pharmacy records describing their lifetime stimulant use. No distinction was made between different stimulant drugs. Permission could not be obtained from 39 participants (17 ADHD; 7 SIBS; 15 CON). For all other participants, medication records were acquired. Records confirmed that no control participants had a history of stimulant use. A few unaffected siblings had a history of stimulant use: 5 siblings had used stimulants up to 2 years, whereas 3 had used stimulants for more than 2 years. It is important to note that these siblings were not on medication around the time of testing. Amongst the 119 participants with ADHD, 102 had a history of stimulant use (with 22 having used/using stimulants for up to 2 years, and 80 having used/using stimulants for more than 2 years). Fourteen participants with ADHD were stimulant drug-naive.

### Behavioural analysis of the MID

Reaction times (RT) for both reward and non-reward conditions were transformed by a log10 transformation to conform to the equality of variance assumption. Trials with responses faster than 100 ms were excluded. RT for Reward and Non-Reward Hits were first compared across all participants in a paired *t* test using SPSS (version 21, IBM Corporation, Armonk, New York, USA). Subsequently, linear mixed models were run in SPSS to model the effects of various factors on the RT for Reward Hits, Non-Reward Hits and the Reward Hit–Non-Reward Hit RT difference. Models included the relevant RT as its dependent variable and used dimensional scores of ASD and ADHD symptoms and their interaction, age, sex, IQ and scan site as fixed effects with Family ID (to control for familial effects) modeled as a random effect. The ASD × ADHD interaction term was calculated as the element-wise multiplication of the centered ASD and ADHD variables. Medication use (cumulative stimulant medication duration) was added as an additional random effect in a separate model that included data from all participants for whom medication data was available. This model served as a sensitivity analysis to investigate the influence of medication usage.

### MRI acquisition

Functional magnetic resonance imaging (fMRI) data was recorded at two separate scan sites using nearly identical acquisition parameters. At the VU University in Amsterdam, data was acquired on a 1.5 T Siemens Sonata scanner; at the Donders Institute for Brain, Cognition and Behaviour in Nijmegen, data was acquired on a 1.5 T Siemens Avanto scanner (both: Siemens Medical, Erlangen, Germany). Whole-brain T2*-weighted images were acquired using an echo planar imaging (EPI) sequence (37 slices in Nijmegen/38 slices in Amsterdam, repetition time = 2340 ms, echo time = 40 ms, field of view = 224 × 224 mm, voxel size = 3.5 × 3.5 × 3.0 mm, matrix = 64 × 64, slice thickness = 3 mm, 17 % gap). Whole-brain T1-weighted anatomical images were acquired at both sites using a magnetization-prepared, rapid acquisition gradient echo (MPRAGE) sequence (176 slices, repetition time = 2730 ms, echo time = 2.95 ms, inversion time = 1000 ms, voxel size = 1.0 × 1.0 × 1.0 mm, field of view = 256 mm). To control for site effects in the neuroimaging data, analyses included scan site as a covariate of no interest.

### MRI preprocessing

Functional and structural imaging data were preprocessed and analysed using the FMRIB Software Library (FSL, version 5 [42]). The first 5 functional volumes of each participant were discarded to allow for T1 equilibrium. All other volumes were realigned to the first remaining volume to correct for head motion. The resulting extended realignment parameters plus the extracted time courses of regions containing white matter and cerebral spinal fluid were then used for nuisance regression. Subsequently, images were spatially smoothed using a Gaussian kernel with a full width at half maximum (FWHM) of 6 mm and high-pass filtered at 0.001 Hz.

Functional images were spatially co-registered to their associated structural image using FSL FLIRT and normalized to MNI152 standard space after first-level statistics had been performed. Considering the wide age range of our sample, we opted to register all participants’ brains to a custom study template that was generated by averaging all T1-scans of participants in the NeuroIMAGE study (*n* = 787), with a resolution of 2 × 2 × 2 mm after transforming it non-linearly to MNI152 space with FSL FNIRT. For each participant, a non-linear warp-field for normalization from T1 to the custom template was calculated and subsequently applied. This procedure minimized the bias towards adult brains and provided a better brain registration for younger participants.

### MRI first-level analysis

For every subject, statistical parametric maps were estimated using a general linear model that included all relevant features of the MID trials (FSL FEAT). Six regressors of interest were included, containing the onsets for reward cues, non-reward cues, reward hits, reward misses, non-reward hits and non-reward misses, with all events modeled with a duration of zero. In addition, six regressors of no interest were included. These regressors modeled 1) movement artifacts; 2,3) the onsets of target presentation for reward and non-reward trials; and 4,5,6) the onsets for the cue, target and outcome of error trials. Movement artifacts were head movements from one image to the next that exceeded a threshold of 0.5 mm in any direction. Event onset of these artifacts was set to 8 s before the movement and all events of interest within this 8 s interval were discarded [43]. Error trials were trials with premature responses (RT <100 ms), too many responses (>1 button press) or no response. All these regressors were modeled including their temporal derivatives and subsequently convolved with a canonical hemodynamic response function (HRF).

The first-level models of the MID task provided two contrasts of interest. The neural effect of reward anticipation was obtained by contrasting BOLD activity evoked by reward and non-reward cues (Reward Cue > Non-Reward Cue). Reward outcome-related activity was quantified by contrasting the effect of correct responses during reward trials with that observed during non-reward trials ([Reward Hit–Reward Miss] > [Non-Reward Hit–Non-Reward Miss]). Estimated beta maps for both contrasts were normalized to MNI152 standard space for each participant for subsequent group comparisons.

### MRI second-level analysis

Group-level analyses modeled neural activation across the full sample of participants (thus including participants with ADHD, their siblings and control participants in the same model). Second-level activation maps were calculated with FSL FLAME using the normalized beta maps from the first-level analyses. Neural responses during reward anticipation and reward outcome were modeled separately at the second level and included the first-level variance estimates to account for between-subject differences in the quality of parameter estimation. The second-level model included the participant-specific ASD symptoms, ADHD symptoms, the ASD × ADHD interaction (the element-wise multiplication of the previous two variables), age, sex, IQ, scan site and ODD/CD comorbidity as explanatory variables (EVs). The factor Group (i.e. ADHD, siblings or control) was only present as EV in post hoc sensitivity analyses. Additional models including separate regressors for ADHD hyperactive/impulsive and inattentive symptoms were run as supplementary analyses (Additional file 4). All EVs were demeaned (using the overall sample mean) before inclusion. The second-level models were calculated using the FSL FLAMEO command and included automatic detection and de-weighting of outliers [44].

### Statistical thresholding

All results reported were based on an initial uncorrected voxel-level threshold of *Z* > 2.3, corrected for the whole brain at the cluster level using *p* < 0.025 (FWE, corrected for testing both reward anticipation and reward receipt).

### Post hoc analyses of second-level MRI results

The mean time-series of each cluster that survived cluster-level correction were extracted for each participant using FSL for post hoc analyses in SPSS. In these analyses, results were corrected for familiality (i.e. the non-independence of data from participants belonging to the same family due to shared genetic and environmental influences). Time-series for each cluster were entered as the dependent variable in a linear mixed model that included ASD symptoms, ADHD symptoms, the ASD × ADHD interaction, age, site and sex as fixed effects and Family ID as a random effect. Moreover, this analysis was repeated for the subset of participants for whom medication data was available, additionally including their total stimulant use duration as a random effect to control for the effect of medication use. This extra model served as a sensitivity analysis to investigate whether our findings were influenced by medication usage. Finally, the presence of interactions between any CSBQASD or CPRS-R-L effect and experimental group (ADHD, SIBS or CON) was checked by running separate models that included Group and a CSBQASD by Group interaction in addition to all previous fixed and random effects. These models served to test whether the observed effects of ASD and ADHD symptoms and the parameter estimates in the regions under investigation differed in the three experimental groups. Alpha was set at *p* = 0.05 for all post hoc analyses.

## Results

### Demographics

Table 1 lists the demographics of the experimental sample. Participants with ADHD were of similar age compared to their siblings and healthy controls (ADHD vs SIBS *t*_217_ = 1.33, *p* = 0.186; ADHD vs CON *t*_239_ = 1.35, *p* = 0.179); unaffected siblings were older than controls (SIBS vs CON, *t*_186_ = 2.32, *p* = 0.022). IQ was similar in participants with ADHD and their siblings (ADHD vs SIBS, *t*_217_ = 0.36, *p* = 0.725), but was higher in controls than the two other groups (CON vs ADHD, *t*_239_ = 5.04, *p* < 0.001; CON vs SIBS, *t*_186_ = 4.22, *p* < 0.001). Scores on the CPRS-R-L were highest for ADHD, as expected (ADHD vs SIBS, *t*_217=_16.58, *p* < 0.001; ADHD vs CON, *t*_239_ = 20.47, *p* < 0.001), but were also elevated in siblings compared to controls (SIBS vs CON, *t*_186_ = 2.75, *p* = 0.007). Finally, CSBQASD scores were higher in the ADHD group and their siblings compared to controls (ADHD vs CON, *t*_239_ = 7.73, *p* < 0.001; SIBS vs CON, *t*_186_ = 4.79, *p* < 0.001), but not significantly different between the former groups (ADHD vs SIBS, *t*_217_ = 1.03, *p* = 0.305). In summary, participants with ADHD showed on average the highest severity of both ADHD and ASD, their unaffected siblings were similar to the ADHD group in ASD but not ADHD severity, and the healthy controls scored lowest on both ADHD and ASD dimensions.

### Behavioural analysis of the MID task

Correct responses were faster for Reward versus Non-Reward Hits (Reward Hit RT ± standard error of the mean (SEM) = 287.41 ± 3.1 ms; Non-Reward Hit RT ± SEM = 317.18 ± 3.8 ms; paired *t* test on the log transformed data: *t*_323_ = −12.73, *p* < 0.001). Subsequent mixed model analyses (controlling for age, IQ, sex, scan site, familial effects and ADHD × ASD interactions) showed no significant association between the CSBQASD or CPRS-R-L scores and any RT measure (Reward Hits RT, Non-Reward Hits RT, or the RT difference between correct Reward and Non-Reward trials, all *p* > 0.05). In summary, although we found that reward trials showed the expected speeding of responses for all participants, we found no evidence that ASD or ADHD symptoms modulated the behavioural response of our participants in the MID task.

### fMRI analysis of reward anticipation

**Fig. 1:**
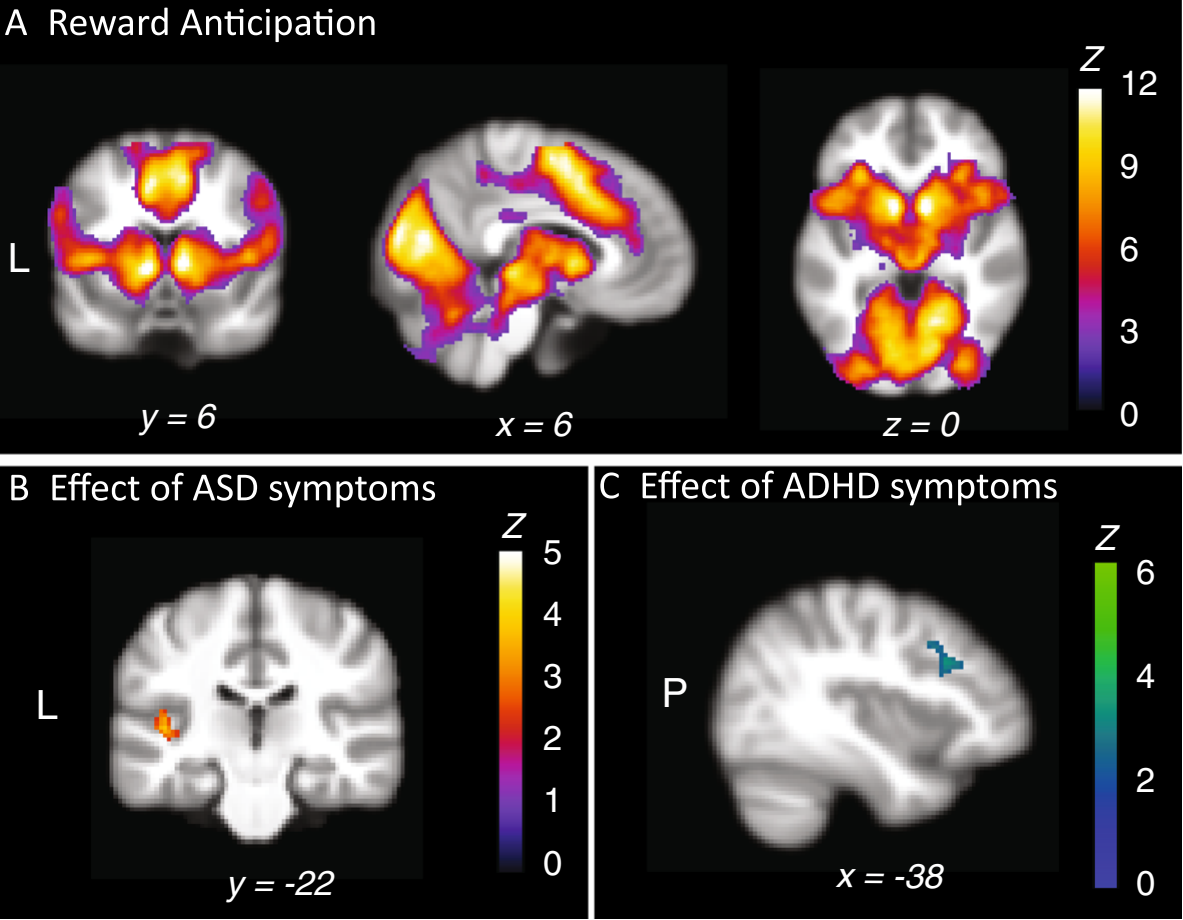
Neural responses associated with reward anticipation. **a** Reward anticipation: activation stronger for Reward versus Non-Reward Cues. Activation plotted represents the linear contrast between reward and non-reward cues from the time of cue onset. Reward anticipation was associated with stronger response in a network of brain areas including the striatum, medial (pre)frontal cortex, bilateral insula and parahippocampus, as well as posterior occipital and parietal regions. **b** ASD symptom scores were positively correlated with left insula activity during reward anticipation. **c** ADHD symptom scores were negatively correlated with left dorsolateral prefrontal cortex activation during reward anticipation. All activation shown was initially thresholded at the voxel level at *Z* > 2.3, followed by whole-brain correction at the voxel level at *p* < 0.025 (FWE). The clusters shown in panels **b** and **c** are significant after correction for familial non-independence and medication use. Results are plotted on representative slices of the NeuroIMAGE study template brain; coordinates are given in MNI space. See Additional file 5 for coordinates, *p* values and cluster extent. *L* left. *P* posterior. *Z Z* value

All fMRI analyses were controlled for effects of age, sex, IQ, scan site and ODD/CD comorbidity. Reward anticipation (Reward Cue > Non-Reward Cue) was associated with significant activation in a network of brain areas including the ventral striatum, amygdala, insula, cingulate cortex and visual areas (Fig. 1a, Additional file 5). Non-reward anticipation (Non-Reward Cue > Reward Cue) was related to stronger activity in the posterior cingulate and bilateral inferior parietal cortex.

ASD scores were positively correlated with activity in bilateral insula and the left superior frontal gyrus during reward anticipation. However, only the association between left insula activity and ASD scores remained significant after correction for familial non-independence and medication use (Fig. 1b, Additional file 5). This effect persisted when restricting our analysis to participants below 18 years of age and was not significantly different in adults and children (Additional file 6). Moreover, we found no significant effect of Group (ADHD, siblings or controls) on left insula activity or on the effect of ASD symptoms in the left insula (Additional file 7). In contrast, ADHD symptoms were negatively correlated with activity in posterior parietal and left dorsolateral prefrontal cortex (dlPFC) during reward anticipation. This negative correlation remained significant in the dlPFC after correction for familial non-independence and medication use (Fig. 1c, Additional File 5). This effect of ADHD symptoms was significant in participants below and above the age of 18 years when analysed separately and was not significantly different in these age groups (Additional file 6). No significant interactions between ASD and ADHD effects on reward anticipation were found. Again, we found no significant effect of Group on left dlPFC activity or on the effect of ADHD symptoms in the dlPFC (Additional file 7). Supplementary analyses looking at the distinct impact of ADHD hyperactive/impulsive and inattentive symptoms showed that the former was associated with reduced parahippocampal and lingual gyrus activity and the latter with reduced caudate activity during reward anticipation (Additional file 4).

### fMRI analysis of reward outcome

**Fig. 2:**
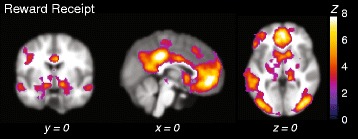
Reward outcome: activation stronger for Rewarded versus Non-Rewarded Outcomes. Activation plotted represents the linear contrast (Reward Hit–Reward Miss) > (Non-Reward Hit–Non-Reward Miss). Reward outcome was associated with increased activity in the striatum, orbito-frontal and prefrontal cortex; bilateral posterior and inferior parietal cortex; posterior, mid and anterior cingulate gyrus and bilateral amygdala and hippocampus. No significant increases in activation were observed for non-rewarded outcomes. All activation shown was initially thresholded at the voxel level at *Z* > 2.3, followed by whole-brain correction at the voxel level at *p* < 0.025 (FWE). Results are plotted on representative slices of the NeuroIMAGE study template brain; coordinates are given in MNI space. See Additional file 5 for coordinates, *p* values and cluster extent. *Z Z* value

The neural response to reward outcome was investigated using the contrast between Rewarded and Non-Rewarded outcomes (Reward Hit-Miss > Non-Reward Hit-Miss). Rewarded hits were associated with significantly stronger activation than non-rewarded hits in the ventral striatum, anterior cingulate and orbito-frontal cortex, posterior cingulate and parietal cortex and posterior visual areas (Fig. 2). Non-rewarded outcomes were not linked to significant increases in activation compared to rewarded outcomes. No significant associations were found between ASD or ADHD scores and neural responses during reward receipt. Supplementary analyses related to distinct effects of ADHD hyperactive/impulsive and inattentive symptoms similarly did not result in significant findings.

## Discussion

In this study, we present evidence that variation in ASD and ADHD symptoms is related to specific changes in the neural signatures of reward processing in patients with ADHD, their unaffected siblings and healthy controls. We found that ASD symptoms were positively related to left insula activity during reward anticipation across the three experimental groups. In contrast, ADHD symptoms were negatively related to activity in left dlPFC during reward anticipation. Both findings could not be explained by effects of age or sex. No effects of either ASD or ADHD were found during reward outcome.

Neural hyperactivity during the processing of reward in ASD is not a common finding, but has been demonstrated previously [19, 18, 16]. Our findings are in line with previous findings by Cascio and colleagues, who also found ASD-related hyperactivity in the insula during reward anticipation [19]. Our results also match well with the proposed role of the insula in interoceptive and motivational processes [45–47] and its theorized relevance to decision-making and abnormal reward-seeking behaviour [48]. Anatomical evidence suggests that ASD is characterized by structural abnormalities in the insula that could relate to heightened interoception, and/or a more internally oriented focus [49, 50]. Taken together, increased insula activity in individuals with higher levels of ASD symptoms might be related to altered motivational processes and/or greater interoception. As such, activity in the insula might be considered a possible marker of cognitive dysfunction in milder forms of ASD.

ADHD symptoms were associated with reduced left dlPFC activity during reward anticipation. ADHD has previously been linked to hypoactivity during anticipation of monetary reward, although primarily in striatal regions [21, 11, 51, 52]. Although the dlPFC is not considered a central part of the neural reward circuit, in our study it was involved in reward anticipation (i.e. more strongly activated during anticipation of reward versus no reward across the sample). This finding would suggest that the dlPFC is differentially responsive to rewarded versus non-rewarded contexts. Furthermore, the dlPFC has shown ADHD-related abnormalities in various cognitive contexts, due to its proposed role in attentional and motivational processes, and our finding could thus reflect more general neurocognitive alterations associated with ADHD [53–55]. In addition, exploratory analyses of the reward anticipation phase using separate ADHD subscales indicated that hyperactive/impulsive symptoms were associated with reduced activity in the parahippocampal and lingual gyrus, whereas inattentive symptoms were linked to reduced caudate activity. These findings provide initial evidence that these subscales might modulate the neural response to reward anticipation differentially.

Against expectation, we found no evidence for striatal effects of ASD symptoms in our study. Although striatal deficits related to reward processing have been observed in multiple ASD studies [51], other studies did not find striatal abnormalities in monetary reward conditions [13, 14, 56]. We can only speculate about the reasons why striatal functioning was unaffected by ASD symptoms in our study. Our large sample size provided enough power to detect effects. Instead, differences in task parameters (e.g. dimensional measures versus categorical definitions of ASD; differences in reward probability and amount of reward) could provide an explanation. In addition, striatal abnormalities might be characteristic of clinical ASD only, and not clearly apparent in less affected populations. Note that we did find that the ventral striatum and other areas of the frontal-striatal reward circuit were robustly activated during reward anticipation and receipt, in line with other studies, indicating that our task manipulation was successful [57, 30].

We did not find evidence for effects of ASD or ADHD symptoms during reward receipt, in the outcome phase of the MID task. Although it is difficult to speculate about the reasons for a null-finding, it could be in part due to the lower number of trials available for the outcome condition. However, we believe that our large sample offers substantial protection against this potential problem, and we do find strong reliable reward outcome-related neural responses. It could nevertheless be that dimensional effects of ADHD and ASD during the reward outcome phase are smaller than those during reward anticipation.

In addition to the expected positive effects of reward anticipation in fronto-striatal regions, we also observed significant activation differences in the reversed contrast (i.e. stronger activity for anticipation of no reward versus reward). These effects were localized in brain areas previously associated with the so-called default mode network and could therefore reflect reduced task engagement (and increased mind-wandering) during non-reward versus reward anticipation [58].

The results of this study should be seen in the context of some strengths and limitations. First, although a large part of the autistic spectrum was covered in our experiment due to the inclusion of three groups of participants with varying degrees of ASD symptoms, we did not measure the extreme end of the spectrum by including participants with a clinical diagnosis of ASD. This makes it difficult to translate our findings to more severely affected populations. However, our results remain relevant for individuals with ADHD and the general population, where milder forms of ASD are commonly observed. To extend our findings and provide converging evidence for the results of the current study, we are currently conducting research using participants with a clinical diagnosis of ASD who are also evaluated for ADHD symptomatology in the EU-AIMS project (www.eu-aims.eu).

We did not find behavioural effects of ASD or ADHD on the MID task in this study. We can therefore only speculate on the behavioural relevance of the current findings. Nevertheless, the absence of behavioural effects also erases a potential confound for the interpretation of the neuroimaging findings, and suggests that both ASD and ADHD symptoms are affecting the neural substrates of reward processing in a way that cannot simply be explained by differences in behaviour. This notwithstanding, the direct clinical relevance of our findings is not immediately apparent and will require future study.

An important strength of the current study was the use of dimensional measures of ADHD and ASD symptoms. These measures allowed for a more refined analytic approach relative to traditional categorical comparisons between populations. Similar approaches are becoming more and more common in psychiatry, as the relevance of dimensional aspects of many psychiatric disorders becomes increasingly apparent [59, 60]. Future studies in ASD and ADHD populations could therefore benefit from including similar designs to further disentangle the contribution of ASD and ADHD symptoms to reward processes.

Unfortunately, this study could not investigate whether ASD (and ADHD) symptoms affect social (and other types of) reward differently from monetary reward. Further research is needed to investigate the specificity of our findings. Follow-up research could clarify whether social reward paradigms show similar effects. Given the ongoing discussion about the special relevance of social reward deficits in ASD and ADHD, such studies could provide valuable novel insights [20, 9].

Since data were available from participants with ADHD (who scored high on ADHD and ASD), their unaffected siblings (who scored high on ASD but not ADHD) and healthy controls (who scored low on ADHD and ASD), our sample included a wide range of ASD and ADHD symptoms. This design, in combination with our large sample size, enabled us to separate effects of ADHD and ASD and study whether both symptom dimensions interacted. We found no evidence for an interaction between ADHD and ASD symptom scores, nor did we find that the neural effects of ASD and ADHD symptoms differed in the three experimental groups. Our findings therefore suggest that ASD and ADHD symptoms affected all types of participants equally (at a given level of severity) and that ASD and ADHD did not have multiplicative effects in our sample. Practically, this would mean that ASD and ADHD affect reward processing in distinct ways and via different (neural) mechanisms. Our findings thus do not directly support theories of a shared aetiology between ASD- and ADHD-related reward dysfunction, nor do they point towards a qualitative difference in reward abnormalities in individuals who score high on both ASD and ADHD symptom measures. However, our sample did not include individuals with clinical levels of both ADHD and ASD, so we cannot rule out that such individuals would show specific abnormalities in line with previous studies [8, 27, 28].

## Conclusions

With this study, we provide evidence that variation in ASD- and ADHD-related symptomatology can modulate the neural response to reward anticipation in participants with ADHD, their unaffected siblings and healthy controls. Taken together, these results underscore the importance of multidimensional assessment for clinical and healthy populations in general and for the characterization of ADHD- and ASD-related effects on reward processing in particular.

## Additional files

Additional file 1:
**Exclusion flowchart.** Details the ex- and inclusion procedure for the current study. (PDF 497 kb) Additional file 2:
**Demographics of participants excluded for motion or too few MID trials.** Lists the demographics of participants excluded for motion or too few MID trials and compares these with those of included participants. (PDF 344 kb) Additional file 3:
**Algorithm for ADHD diagnosis in NeuroIMAGE.** Describes the diagnostic algorithm for assessment of ADHD in NeuroIMAGE. (PDF 531 kb) Additional file 4:
**Analyses of hyperactive/impulsive and inattentive symptoms.** Describes the methods and results of supplementary analyses of the effects of hyperactive/impulsive and inattentive symptoms on the neural response to reward processes. (PDF 346 kb) Additional file 5:
**Table of MRI results.** Includes additional information about the main MRI results of the current study. (PDF 230 kb) Additional file 6:
**Comparison of effects in Adults and Participants under 18.** Includes additional information about and results from the comparison of ADHD and ASD effects in adults and participants under 18. (PDF 485 kb) Additional file 7:
**Plots of ASD and ADHD effects in the three experimental groups.** Contains plots of the effects of ASD and ADHD on reward anticipation split across the three experimental groups. (PDF 317 kb)

## Abbreviations

ABR: Algemeen Beoordelings en Registratieformulier *(=General form for evaluation and registration of experimental studies)*
ADHD: attention-deficit/hyperactivity disorder
ASD: autism spectrum disorder
bil: bilateral
BOLD: blood oxygen level dependent
CAARS-O:SV: Conners Adult ADHD Rating Scales–Observer: Screening Version
CD: conduct disorder
CMO: Commissie Mensgebonden Onderzoek (=*Committee for Research in Humans; medical-ethical committee)*
CON: controls
CPRS-R-L: Conners Parent Rating Scales Revised: Long Version
CSBQ: Children’s Social Behavioral Questionnaire
CTRS-R-L: Conners Teacher Rating Scales Revised: Long Version
dlPFC: dorsolateral prefrontal cortex
DSM: Diagnostic and Statistical Manual of Mental Disorders
EFPIA: European Federation of Pharmaceutical Industries and Associations
EPI: echo planar imaging
EU-AIMS: European Autism Interventions–A Multicentre Study for Developing New Medications
EV: explanatory variable
FEAT: FMRI Expert Analysis Tool
FLIRT: FMRIB Linear Image Registration Tool
fMRI: functional magnetic resonance imaging
FNIRT: FMRIB Nonlinear Image Registration Tool
FSL: FMRIB Software Library
FWE: familywise error
FWHM: full width at half maximum
HRF: hemodynamic response function
Incl: included
IQ: intelligence quotient
KSADS-PL: Kiddie-Sads-Present and Lifetime version
M: male
MID: monetary incentive delay
MNI: Montreal Neurological Institute
mPFC: medial prefrontal cortex
MPRAGE: magnetization-prepared rapid acquisition gradient echo
MR: magnetic resonance
MRI: magnetic resonance imaging
ms: millisecond
NA: not applicable
NeuroIMAGE: Neuroimaging part of the International Multisite ADHD Genetics project
NIH: National Institutes of Health
NS: not significant
NWO: Nederlandse organisatie voor Wetenschappelijk Onderzoek *(=Dutch Organization for Scientific Research)*
ODD: oppositional defiant disorder
RT: reaction time
SD: standard deviation
SEM: standard error of the mean
SIBS: siblings
SPSS: Statistical Package for the Social Sciences
USA: United States of America
ZonMW: Zorginnovatie en Medische Wetenschappen *(=Healthcare Innovation and Medical Sciences*)

## References

[1] American Psychiatric Association (2013). Diagnostic and Statistical Manual of Mental Disorders.

[2] Constantino JN, Todd RD (2003). Autistic traits in the general population: a twin study. Arch Gen Psychiatry.

[3] Constantino JN, Todd RD (2005). Intergenerational transmission of subthreshold autistic traits in the general population. Biol Psychiatry.

[4] Ronald A, Happe F, Price TS, Baron-Cohen S, Plomin R (2006). Phenotypic and genetic overlap between autistic traits at the extremes of the general population. J Am Acad Child Adolesc Psychiatry.

[5] Mulligan A, Anney RJ, O'Regan M, Chen W, Butler L, Fitzgerald M (2009). Autism symptoms in attention-deficit/hyperactivity disorder: a familial trait which correlates with conduct, oppositional defiant, language and motor disorders. J Autism Dev Disord.

[6] Reiersen AM, Constantino JN, Volk HE, Todd RD (2007). Autistic traits in a population-based ADHD twin sample. J Child Psychol Psychiatry.

[7] Taurines R, Schwenck C, Westerwald E, Sachse M, Siniatchkin M, Freitag C (2012). ADHD and autism: differential diagnosis or overlapping traits? A selective review. Atten Defic Hyperact Disord.

[8] van der Meer JM, Oerlemans AM, van Steijn DJ, Lappenschaar MG, de Sonneville LM, Buitelaar JK (2012). Are autism spectrum disorder and attention-deficit/hyperactivity disorder different manifestations of one overarching disorder? Cognitive and symptom evidence from a clinical and population-based sample. J Am Acad Child Adolesc Psychiatry.

[9] Rommelse NN, Geurts HM, Franke B, Buitelaar JK, Hartman CA (2011). A review on cognitive and brain endophenotypes that may be common in autism spectrum disorder and attention-deficit/hyperactivity disorder and facilitate the search for pleiotropic genes. Neurosci Biobehav Rev.

[10] Banaschewski T, Poustka L, Holtmann M (2011). Autism and ADHD across the life span. Differential diagnoses or comorbidity?. Nervenarzt.

[11] Kohls G, Thonessen H, Bartley GK, Grossheinrich N, Fink GR, Herpertz-Dahlmann B (2014). Differentiating neural reward responsiveness in autism versus ADHD. Dev Cogn Neurosci.

[12] Dichter GS (2012). Functional magnetic resonance imaging of autism spectrum disorders. Dialogues Clin Neurosci.

[13] Scott-Van Zeeland AA, Dapretto M, Ghahremani DG, Poldrack RA, Bookheimer SY (2010). Reward processing in autism. Autism Res.

[14] Delmonte S, Balsters JH, McGrath J, Fitzgerald J, Brennan S, Fagan AJ (2012). Social and monetary reward processing in autism spectrum disorders. Mol Autism.

[15] Richey JA, Rittenberg A, Hughes L, Damiano CR, Sabatino A, Miller S (2013). Common and distinct neural features of social and non-social reward processing in autism and social anxiety disorder. Soc Cogn Affect Neurosci.

[16] Dichter GS, Felder JN, Green SR, Rittenberg AM, Sasson NJ, Bodfish JW (2012). Reward circuitry function in autism spectrum disorders. Soc Cogn Affect Neurosci.

[17] Kohls G, Schulte-Ruther M, Nehrkorn B, Muller K, Fink GR, Kamp-Becker I (2013). Reward system dysfunction in autism spectrum disorders. Soc Cogn Affect Neurosci.

[18] Dichter GS, Richey JA, Rittenberg AM, Sabatino A, Bodfish JW (2012). Reward circuitry function in autism during face anticipation and outcomes. J Autism Dev Disord.

[19] Cascio CJ, Foss-Feig JH, Heacock JL, Newsom CR, Cowan RL, Benningfield MM (2012). Response of neural reward regions to food cues in autism spectrum disorders. J Neurodev Disord.

[20] Chevallier C, Kohls G, Troiani V, Brodkin ES, Schultz RT (2012). The social motivation theory of autism. Trends Cogn Sci.

[21] Plichta MM, Scheres A (2013). Ventral-striatal responsiveness during reward anticipation in ADHD and its relation to trait impulsivity in the healthy population: a meta-analytic review of the fMRI literature. Neurosci Biobehav Rev.

[22] Rubia K, Halari R, Cubillo A, Mohammad AM, Brammer M, Taylor E (2009). Methylphenidate normalises activation and functional connectivity deficits in attention and motivation networks in medication-naive children with ADHD during a rewarded continuous performance task. Neuropharmacology.

[23] Paloyelis Y, Mehta MA, Faraone SV, Asherson P, Kuntsi J (2012). Striatal sensitivity during reward processing in attention-deficit/hyperactivity disorder. J Am Acad Child Adolesc Psychiatry.

[24] Gatzke-Kopp LM, Beauchaine TP, Shannon KE, Chipman J, Fleming AP, Crowell SE (2009). Neurological correlates of reward responding in adolescents with and without externalizing behavior disorders. J Abnorm Psychol.

[25] Strohle A, Stoy M, Wrase J, Schwarzer S, Schlagenhauf F, Huss M (2008). Reward anticipation and outcomes in adult males with attention-deficit/hyperactivity disorder. Neuroimage.

[26] Chantiluke K, Barrett N, Giampietro V, Brammer M, Simmons A, Murphy DG (2015). Inverse effect of fluoxetine on medial prefrontal cortex activation during reward reversal in ADHD and autism. Cereb Cortex.

[27] Chantiluke K, Christakou A, Murphy CM, Giampietro V, Daly EM, Ecker C (2014). Disorder-specific functional abnormalities during temporal discounting in youth with attention deficit hyperactivity disorder (ADHD), autism and comorbid ADHD and autism. Psychiatry Res.

[28] Nyden A, Niklasson L, Stahlberg O, Anckarsater H, Wentz E, Rastam M (2010). Adults with autism spectrum disorders and ADHD neuropsychological aspects. Res Dev Disabil.

[29] O'Dwyer L, Tanner C, van Dongen EV, Greven CU, Bralten J, Zwiers MP (2014). Brain volumetric correlates of autism spectrum disorder symptoms in attention deficit/hyperactivity disorder. PLoS ONE.

[30] Knutson B, Cooper JC (2005). Functional magnetic resonance imaging of reward prediction. Curr Opin Neurol.

[31] von Rhein D, Mennes M, van Ewijk H, Groenman A, Zwiers M, Oosterlaan J et al. The NeuroIMAGE study: a prospective phenotypic, cognitive, genetic and MRI study in children with attention-deficit/hyperactivity disorder. Design and descriptives. European Child & Adolescent Psychiatry. 2014:1–17. doi:10.1007/s00787-014-0573-4. 10.1007/s00787-014-0573-4 25012461

[32] von Rhein D, Cools R, Zwiers MP, van der Schaaf M, Franke B, Luman M et al. Increased neural responses to reward in adolescents and young adults with attention-deficit/hyperactivity disorder and their unaffected siblings. Journal of the American Academy of Child & Adolescent Psychiatry. doi:10.1016/j.jaac.2015.02.012. 10.1016/j.jaac.2015.02.012 PMC4417499 25901776

[33] Knutson B, Fong GW, Adams CM, Varner JL, Hommer D (2001). Dissociation of reward anticipation and outcome with event-related fMRI. Neuroreport.

[34] Hermans EJ, Bos PA, Ossewaarde L, Ramsey NF, Fernandez G, van Honk J (2010). Effects of exogenous testosterone on the ventral striatal BOLD response during reward anticipation in healthy women. Neuroimage.

[35] Ossewaarde L, Verkes RJ, Hermans EJ, Kooijman SC, Urner M, Tendolkar I (2011). Two-week administration of the combined serotonin-noradrenaline reuptake inhibitor duloxetine augments functioning of mesolimbic incentive processing circuits. Biol Psychiatry.

[36] Ossewaarde L, van Wingen GA, Kooijman SC, Backstrom T, Fernandez G, Hermans EJ (2011). Changes in functioning of mesolimbic incentive processing circuits during the premenstrual phase. Soc Cogn Affect Neurosci.

[37] Hartman CA, Luteijn E, Moorlag A, de Bildt A, Minderaa R (2007). Manual for the CSBQ.

[38] Hartman CA, Luteijn E, Serra M, Minderaa R (2006). Refinement of the Children's Social Behavior Questionnaire (CSBQ): an instrument that describes the diverse problems seen in milder forms of PDD. J Autism Dev Disord.

[39] Volkmar FR. Encyclopedia of Autism Spectrum Disorders. Springer; 2012.

[40] Conners CK, Sitarenios G, Parker JD, Epstein JN (1998). The revised Conners' Parent Rating Scale (CPRS-R): factor structure, reliability, and criterion validity. J Abnorm Child Psychol.

[41] Conners CK, Erhardt D, Sparrow EP (1999). Conners' Adult ADHD Rating Scles: CAARS.

[42] Jenkinson M, Beckmann CF, Behrens TE, Woolrich MW, Smith SM (2012). Fsl. Fsl Neuroimage.

[43] Keulers EH, Goulas A, Jolles J, Stiers P (2012). Maturation of task-induced brain activation and long range functional connectivity in adolescence revealed by multivariate pattern classification. Neuroimage.

[44] Woolrich MW, Jbabdi S, Patenaude B, Chappell M, Makni S, Behrens T (2009). Bayesian analysis of neuroimaging data in FSL. Neuroimage.

[45] Critchley HD, Wiens S, Rotshtein P, Ohman A, Dolan RJ (2004). Neural systems supporting interoceptive awareness. Nat Neurosci.

[46] Craig AD (2002). How do you feel? Interoception: the sense of the physiological condition of the body. Nat Rev Neurosci.

[47] Craig AD (2009). How do you feel--now? The anterior insula and human awareness. Nat Rev Neurosci.

[48] Naqvi NH, Bechara A (2010). The insula and drug addiction: an interoceptive view of pleasure, urges, and decision-making. Brain Struct Funct.

[49] Santos M, Uppal N, Butti C, Wicinski B, Schmeidler J, Giannakopoulos P (2011). Von Economo neurons in autism: a stereologic study of the frontoinsular cortex in children. Brain Res.

[50] Allman JM, Tetreault NA, Hakeem AY, Manaye KF, Semendeferi K, Erwin JM (2011). The von Economo neurons in the frontoinsular and anterior cingulate cortex. Ann N Y Acad Sci.

[51] Dichter GS, Damiano CA, Allen JA (2012). Reward circuitry dysfunction in psychiatric and neurodevelopmental disorders and genetic syndromes: animal models and clinical findings. J Neurodev Disord.

[52] Hoogman M, Aarts E, Zwiers M, Slaats-Willemse D, Naber M, Onnink M (2011). Nitric oxide synthase genotype modulation of impulsivity and ventral striatal activity in adult ADHD patients and healthy comparison subjects. Am J Psychiatry.

[53] Arnsten AF (2011). Catecholamine influences on dorsolateral prefrontal cortical networks. Biol Psychiatry.

[54] Arnsten AF, Rubia K (2012). Neurobiological circuits regulating attention, cognitive control, motivation, and emotion: disruptions in neurodevelopmental psychiatric disorders. J Am Acad Child Adolesc Psychiatry.

[55] Sonuga-Barke EJ, Fairchild G (2012). Neuroeconomics of attention-deficit/hyperactivity disorder: differential influences of medial, dorsal, and ventral prefrontal brain networks on suboptimal decision making?. Biol Psychiatry.

[56] Schmitz N, Rubia K, van Amelsvoort T, Daly E, Smith A, Murphy DG (2008). Neural correlates of reward in autism. Br J Psychiatry.

[57] Haber SN, Knutson B (2010). The reward circuit: linking primate anatomy and human imaging. Neuropsychopharmacology.

[58] Raichle ME, Snyder AZ (2007). A default mode of brain function: a brief history of an evolving idea. Neuroimage.

[59] Kraemer HC (2007). DSM categories and dimensions in clinical and research contexts. Int J Methods Psychiatr Res.

[60] Hudziak JJ, Achenbach TM, Althoff RR, Pine DS (2007). A dimensional approach to developmental psychopathology. Int J Methods Psychiatr Res.

