# Supplementary material for: Distinct effects of ASD and ADHD symptoms on reward anticipation in participants with ADHD, their unaffected siblings and healthy controls: a cross-sectional study
Source: Mol Autism. 2015 Aug 28;6:48. doi: 10.1186/s13229-015-0043-y (PMC4551566; doi:10.1186/s13229-015-0043-y)
Supplement: Additional file 1: — Exclusion flowchart. Details the ex- and inclusion procedure for the current study. (PDF 497 kb) [file 13229_2015_43_MOESM1_ESM.pdf]

Additional File 1. Exclusion flowchart

**All NeuroIMAGE participants scanned using the MID paradigm**	**N=564**
**Exclusion of participants due to:**	
Subthreshold, unconfirmed or remittent ADHD diagnosis	**N=63**
Incomplete MID data	**N=17**
Technical problems during data acquisition	**N=22**
Medication use during testing	**N=8**
Acute psychiatric conditions other than ADHD	**N=4**
Scientifically or clinically relevant incidental findings observed in MR data	**N=11**
Excessive motion during MRI recording	**N=16**
Insufficient number of MID trials	**N=82**
Incomplete CSBQ data	**N=17**
**Total excluded**	**N=240**
**Total included**	**N=324**
A. Participants with a confirmed diagnosis of ADHD	**N=136**
B. Unaffected siblings of participants from Category A	**N=83**
C. Unrelated and unaffected control participants	**N=105**
**Subsample of participants included from the sample used by Von Rhein *et al.,* 2015**	
**A.** Participants with a confirmed diagnosis of ADHD	**91%***
**B.** Unaffected siblings of participants from Category A	**90%***
**C.** Unrelated and unaffected control participants	**97%***

*Percentages represent percentage of sample used in Von Rhein *et al*. 2015 that could be included in the current analyses.
