# Supplementary material for: Distinct effects of ASD and ADHD symptoms on reward anticipation in participants with ADHD, their unaffected siblings and healthy controls: a cross-sectional study
Source: Mol Autism. 2015 Aug 28;6:48. doi: 10.1186/s13229-015-0043-y (PMC4551566; doi:10.1186/s13229-015-0043-y)
Supplement: Additional file 2: — Demographics of participants excluded for motion or too few MID trials. Lists the demographics of participants excluded for motion or too few MID trials and compares these with those of included participants. (PDF 344 kb) [file 13229_2015_43_MOESM2_ESM.pdf]

Additional File 2.

Demographics of the participants excluded for excessive motion

	ADHD	Siblings	Control	Comparison*
	N=13	*N=1*	*N=2*	Unequal**
	Mean±SD	Mean	Mean±SD	
Age (years)	13.62±2.82	14.06	12.45±1.42	$ADHD_{excl}$<$ADHD_{incl}$ CON_{excl}<CON_{incl}
IQ	91.54±14.15	112	104.50±14.85	NS
Conners Score (Combined Scales)	30.15±9.48	5	5±4.24	$ADHD_{excl}$>$ADHD_{incl}$
Conners Inattentiveness	16.77±5.21	2	4.5±3.54	NS
Conners Hyperactivity/Impulsivity	13.38±5.82	3	0.5±0.71	$ADHD_{excl}$>$ADHD_{incl}$
CSBQ ASD	10.31±8.91	2	3.5±4.95	NS
CSBQ Lack of Social Interest	3.85±	2	2±3.62	NS
CSBQ Problems with Social Understanding	3.92±	0	1±1.41	NS
CSBQ Stereotypical Behavior	1.38±	0	0±0	NS
CSBQ Resistance to Change	1.15±	0	0.5±0.71	NS
Adult	0%	0%	0%	$ADHD_{excl}$<$ADHD_{incl}$
Site	31%	0 %	50%	NS
Sex	54% M	0% M	50% M	NS

*Comparison of distributions in excluded and included diagnostic groups using Mann-Whitney tests at $p<0.05$. The *Siblings* groups did not show significant differences but results cannot readily be interpreted due to exclusion of only 1 sibling; similar caution should be taken when interpreting the results in the *Control* group (n=2). **Participants with ADHD are significantly overrepresented in the exclusion sample compared to their proportion in the included sample (as tested with a Mann-Whitney test). **SD**=Standard deviation; **ADHD**=participants with ADHD; **SIBS** = unaffected siblings, **CON**= unrelated control participants. **Adult**= % of participants aged 18 years or older. **Site** = % of participants scanned in Amsterdam (the remainder was scanned in Nijmegen). $_{\mathbf{Incl}}$=Included participants; $_{\mathbf{Excl}}$=Excluded participants. **M**=Male. **NS**= not significant.

Demographics of participants excluded for having too few MID trials

	ADHD	Siblings	Control	Comparison*
	N=52	*N=13*	*N=17*	Unequal**
	Mean±SD	Mean	Mean±SD	
Age (years)	15.91±3.43	13.42±2.55	14.61±3.10	$ADHD_{excl}$<$ADHD_{incl}$ $SIBS_{excl}$<$SIBS_{incl}$ CON_{excl}<CON_{incl}
IQ	94.79+16.34	105.77±13.72	107.47±12.40	NS
Conners Score (Combined Scales)	23.63±11.00	5.92±5.81	4.71±4.38	NS
Conners Inattentiveness	13.35±6.11	3.54±3.33	3.47±3.24	NS
Conners Hyperactivity/Impulsivity	10.31±6.20	2.38±2.63	1.24±1.60	NS
CSBQ ASD	10.12±9.53	11.31±9.81	4.12±5.36	NS
CSBQ Lack of Social Interest	3.48±4.42	4.85±4.90	1.29±1.69	NS
CSBQ Problems with Social Understanding	4.46±3.96	3.69±2.70	2.00±2.72	NS
CSBQ Stereotypical Behavior	1.44±1.85	1.15±2.12	0.53±1.38	NS
CSBQ Resistance to Change	0.73±1.09	1.62±2.14	0.29±0.77	NS
Adult	29%	12%	0%	$ADHD_{excl}$<$ADHD_{incl}$ $SIBS_{excl}$<$SIBS_{incl}$ CON_{excl}<CON_{incl}
Site	48 %	54%	35%	NS
Sex	35% M	69% M	47% M	NS

*Comparison of distributions in excluded and included diagnostic groups using Mann-Whitney tests at $p<0.05$. **Participants with ADHD are significantly overrepresented in the exclusion sample compared to their proportion in the included sample (as tested with a Mann-Whitney test). **SD**=Standard deviation; **ADHD**=participants with ADHD; **SIBS** = unaffected siblings, **CON**= unrelated control participants. **Adult**= % of participants aged 18 years or older. **Site** = % of participants scanned in Amsterdam (the remainder was scanned in Nijmegen). $_{\mathbf{Incl}}$=Included participants; $_{\mathbf{Excl}}$=Excluded participants. **M**=Male. **NS**= not significant.
