# Supplementary material for: Distinct effects of ASD and ADHD symptoms on reward anticipation in participants with ADHD, their unaffected siblings and healthy controls: a cross-sectional study
Source: Mol Autism. 2015 Aug 28;6:48. doi: 10.1186/s13229-015-0043-y (PMC4551566; doi:10.1186/s13229-015-0043-y)
Supplement: Additional file 3: — Algorithm for ADHD diagnosis in NeuroIMAGE. Describes the diagnostic algorithm for assessment of ADHD in NeuroIMAGE. (PDF 531 kb) [file 13229_2015_43_MOESM3_ESM.pdf]

Additional File 3. Algorithm for ADHD diagnosis in NeuroIMAGE.

Diagnosis took place as stated on the Neuroimage website; full text (with additional clarifications where necessary) is reprinted below.

Direct link:
http://www.neuroimage.nl/index.php?option=com_content&view=article&id=24&Itemid=53

ADHD Diagnostic Algorithm

To determine psychiatric diagnoses, all participants (children and parents alike) were assessed with a combination of ADHD rating scales and a semi-structured diagnostic interview. In order to determine ADHD diagnoses, a diagnostic algorithm was applied based on the behavioral questionnaires (typically filled in by parents as well as a second observer) and the diagnostic interview, using DSM-IV criteria [1]. Inconsistent cases were reviewed by a team of trained experts, in order to derive a consensus diagnosis. Children were assessed with a parent rating scale (CPRS-R:L;[2]), and either a teacher rating scale (CTRS-R:L; [3]), applied for children < 18 years, or a self-report (CAARS-S:S; [4]), applied for children ≥ 18 years.

A semi-structured diagnostic interview (KSADS-PL; [5]) was administered to both the children (if ≥ 12 years old) and their parents separately. Initially, all participants were only administered the screening interview. Participants with elevated scores on any of the screen items for any disorder were administered the full section for these disorders. Participants in the ADHD group were thus always administered the screening interview plus at least the full ADHD section, and were excluded from the sample if they would classify for diagnosis of any other disorder but ADHD, ODD and/or CD. *Parents* were assessed similarly with an observer ADHD rating scale (CAARS-O:SV; [4]), typically filled in by their partner. The KSADS-PL was administered to all parents, who were, if possible, interviewed together with their partner. Of the Conners' ADHD questionnaires the following scales were used:

- DSM Inattentive behaviour
- DSM Hyperactive/Impulsive behaviour
- DSM Total

For all participants using medication, ratings were done of the participant's functioning off medication.

The diagnostic algorithm

The diagnostic algorithm applied to all participants was based on a combination of symptom counts on the ADHD rating scales and the KSADS-PL, both providing operational definitions of each of the 18 behavioral symptoms of ADHD defined by the DSM-IV. Combined counts for each symptom were determined based on the KSADS-PL scores combined with scores on either the teacher rating scale (for children <18 years), the self-report (for children ≥18), or the observer rating (for parents). Based on the algorithm, participants were given either an 'affected' (ADHD diagnosis) status or 'unaffected' status.

The following criteria were used to classify ADHD ('affected' status):

- Combined symptom count of ≥ 6 symptoms of inattentive or hyperactive/impulsive behaviour
- T-score ≥ 63 on at least one of the ADHD subscales on at least one of the available Conners' ADHD rating scales
- Age of onset before 12
- Symptoms cause clinical impairment
- Symptoms are not better accounted for by another disorder

For children ≥18 years and parents, criteria were slightly adapted, such that a combined symptom count of 5 symptoms and age of onset before 15 years were sufficient for an 'affected' status.

Participants were labeled 'unaffected' if they received a T<63 on each of the scales of the Conners' rating scales, and if they had ≤ 3 symptoms (or ≤ 2 symptoms for children of ≥18 years and parents), derived from the combined symptom counts.

For analysis purposes, participants who did not meet criteria for either affected or unaffected status, were labeled 'subthreshold ADHD'.

References:

1. American Psychiatric Association. Diagnostic criteria from DSM-IV-TR. Washington, D.C.: American Psychiatric Association; 2000.
2. Conners CK, Sitarenios G, Parker JD, Epstein JN. The revised Conners' Parent Rating Scale (CPRS-R): factor structure, reliability, and criterion validity. J Abnorm Child Psychol. 1998;26(4):257-68.
3. Conners CK, Sitarenios G, Parker JD, Epstein JN. Revision and restandardization of the Conners Teacher Rating Scale (CTRS-R): factor structure, reliability, and criterion validity. J Abnorm Child Psychol. 1998;26(4):279-91.
4. Conners CK, Erhardt D, Sparrow EP. Conners' Adult ADHD Rating Scles: CAARS. Tonawanda, NY: Multi-Health Systems; 1999.
5. Kaufman J, Birmaher B, Brent D, Rao U, Flynn C, Moreci P et al. Schedule for Affective Disorders and Schizophrenia for School-Age Children-Present and Lifetime Version (K-SADS-PL): initial reliability and validity data. J Am Acad Child Adolesc Psychiatry. 1997;36(7):980-8.
