# Supplementary material for: Distinct effects of ASD and ADHD symptoms on reward anticipation in participants with ADHD, their unaffected siblings and healthy controls: a cross-sectional study
Source: Mol Autism. 2015 Aug 28;6:48. doi: 10.1186/s13229-015-0043-y (PMC4551566; doi:10.1186/s13229-015-0043-y)
Supplement: Additional file 4: — Analyses of hyperactive/impulsive and inattentive symptoms. Describes the methods and results of supplementary analyses of the effects of hyperactive/impulsive and inattentive symptoms on the neural response to reward processes. (PDF 346 kb) [file 13229_2015_43_MOESM4_ESM.pdf]

Additional File 4. Analyses of hyperactive/impulsive and inattentive symptoms

To further investigate the effects of ADHD symptoms, we conducted supplementary analyses using the raw scores on the Conners DSM Hyperactive/impulsive (ADHD Hyp) and Conners DSM Inattentive (ADHD Inatt) subscales as separate regressors in our group-level analyses.

These analyses modeled neural activation across the full sample of participants (thus including participants with ADHD, their siblings and control participants in the same model). 2nd level activation maps were calculated with FSL FLAME using the normalized beta maps from the 1st level analyses. Neural responses during reward anticipation and reward outcome were modeled separately at the 2nd level, and included the 1st level variance estimates to account for between-subject differences in the quality of parameter estimation. The 2nd level model included the participant-specific ASD symptoms (i.e. the CSBQASD score), ADHD Hyp, ADHD Inatt , Age, Sex, IQ, Scan Site, and ODD/CD comorbidity as explanatory variables (EVs). All EVs were demeaned (using the overall sample mean) before inclusion. The 2nd level models were calculated using the FSL flameo command and included automatic detection and de-weighting of outliers.

The resulting Z-maps were thresholded at $Z>2.3$, and underwent cluster-level correction at $p<0.05$ (FWE). The mean time-series of each cluster that survived cluster-level correction were extracted for each participant using FSL for post-hoc analyses in SPSS. In these analyses, results were corrected for familiality (i.e. the non-independence of data from participants belonging to the same family due to shared genetic and environmental influences). Time-series for each cluster were entered as the dependent variable in a linear mixed model that included ASD Symptoms, ADHD Hyp, ADHD Inatt, the ASD x ADHD Hyp interaction, the ASD x ADHD Inatt interaction, the ADHD Hyp x ADHD Inatt interaction, Age, Site and Sex as fixed effects and Family ID as a random effect. Moreover, this analysis was repeated for the subset of participants for whom medication data was available, additionally including their total stimulant duration as a random effect to control for the effect of medication use. This extra model served as a sensitivity analysis to investigate whether our findings were influenced by medication usage. Alpha was set at $p=0.05$ for all results.

After thresholding and correction via the procedure above, we found that:

- ADHD Hyperactive/impulsive symptoms were associated with decreased parahippocampal and lingual activity during reward anticipation.
- ADHD Inattentive symptoms were associated with decreased bilateral caudate activity during reward anticipation.
- ASD symptoms continued to correlate positively with left insula activity during reward anticipation in this model.

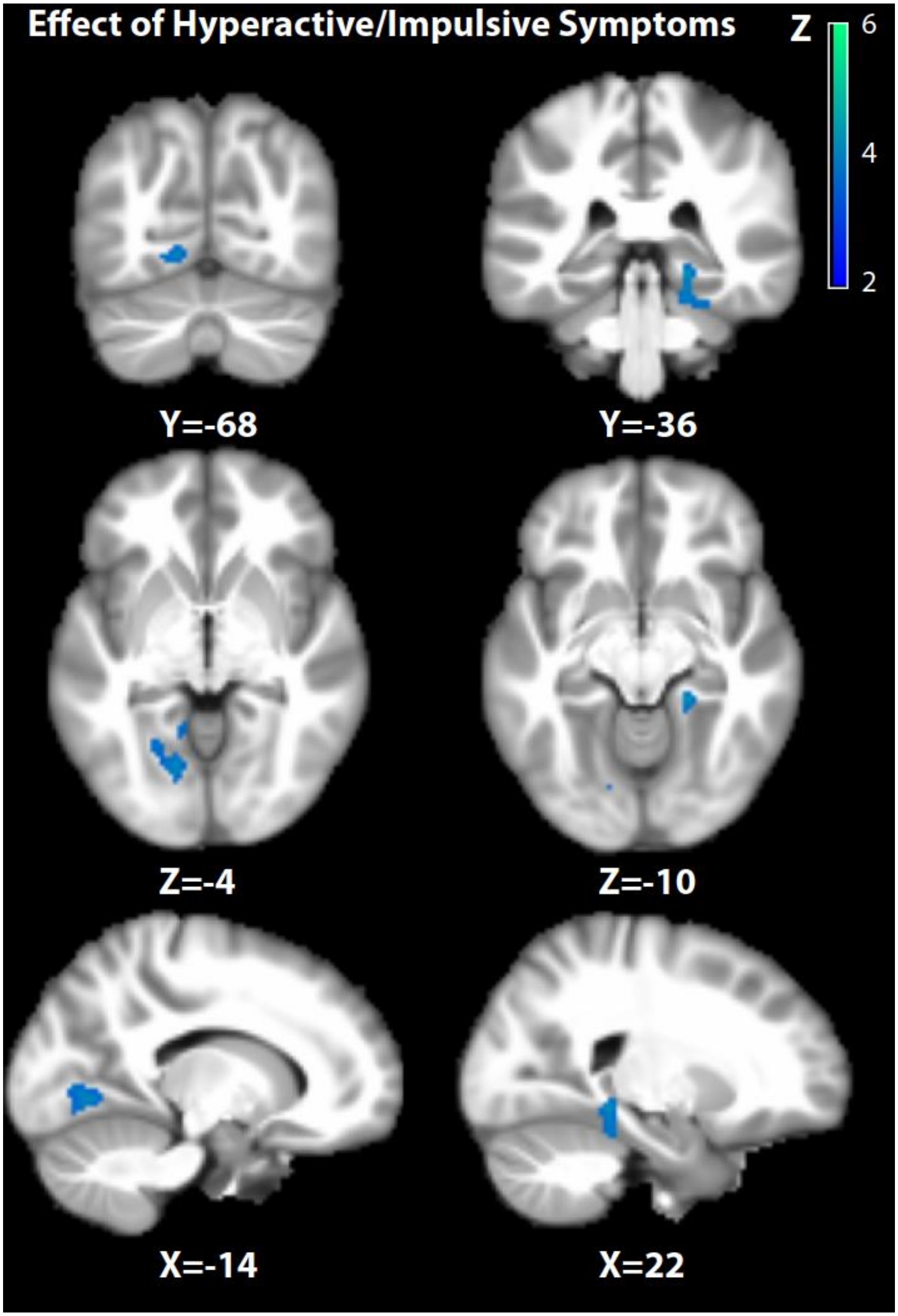

Figure A. Negative effect of Conners DSM Hyperactive/Impulsivity symptoms on the neural response to Reward Anticipation. All activation shown was initially thresholded at the voxel level at Z>2.3, followed by whole-brain correction at the voxel level at p<0.025 (FWE). Results are plotted on representative slices of the NeuroIMAGE study template brain; coordinates are given in MNI space. **Z**= Z score.

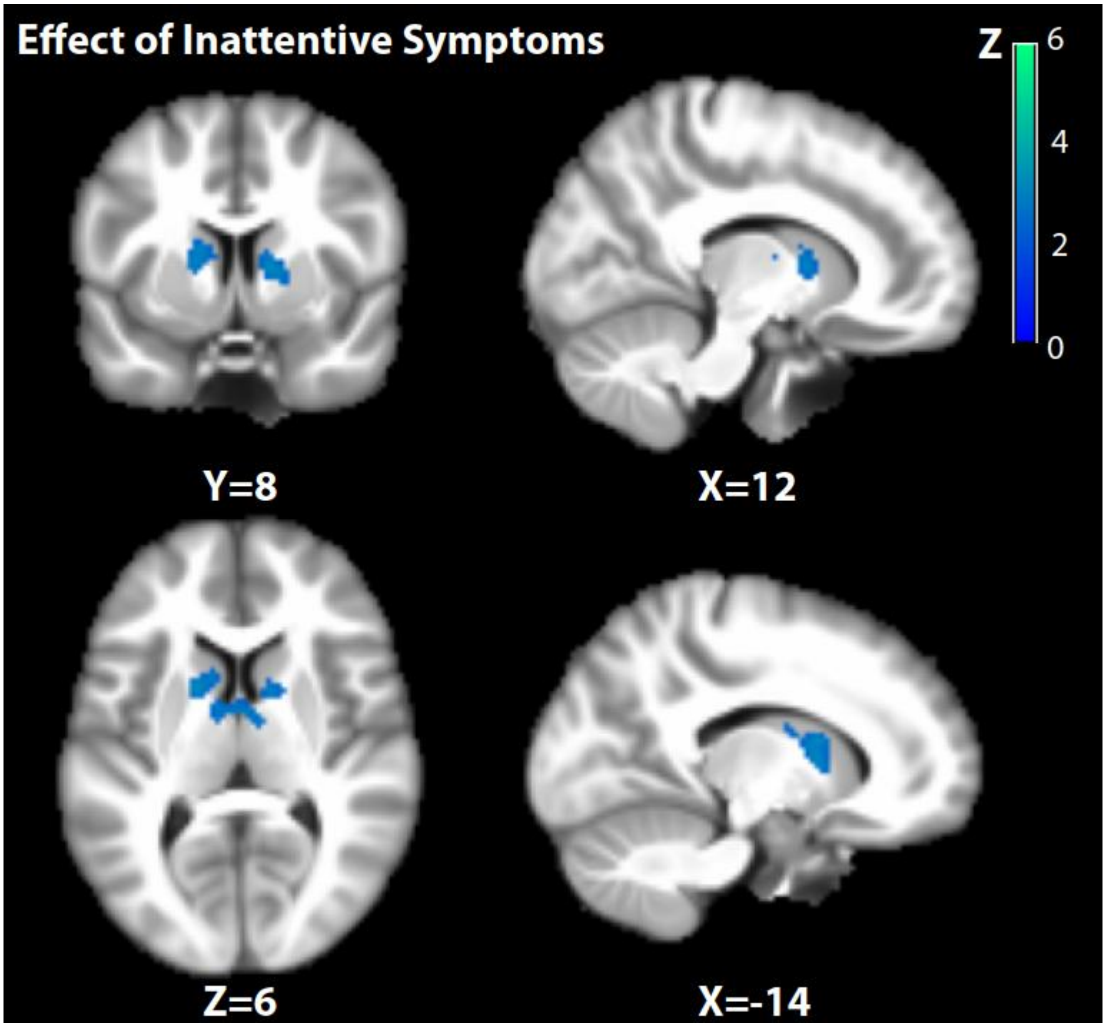

Figure B. Negative effect of Conners DSM Inattentive symptoms on the neural response to Reward Anticipation. All activation shown was initially thresholded at the voxel level at Z>2.3, followed by whole-brain correction at the voxel level at p<0.025 (FWE). Results are plotted on representative slices of the NeuroIMAGE study template brain; coordinates are given in MNI space. **Z**= Z score.

REWARD ANTICIPATION					
Contrast	Cluster location	MNI coordinates* (X Y Z)	Cluster extent (voxels)	Initial p**	Corrected p***
Negative effect of Conners Hyperactive/ Impulsive scale+	Right parahippocampal gyrus	22 -36 -10	86	0.0125	<0.001
	Left lingual gyrus	-14 -68 -4	179	1.14×10^{-5}	0.049
Negative effect of Conners Hyperactive/ Impulsive scale+	Left Caudate	-14 8 14	150	6.3×10^{-5}	<0.001
	Left Thalamus / Caudate	-8 2 8	93	0.0056	0.001
	Right Caudate	14 6 6	80	0.017	0.001
Positive effect of ASD symptoms+	Left Insula	-44 -24 6	215	2.74×10^{-6}	0.001

*MNI coordinates refer to the cluster maximum in MNI space.

**Initial p: cluster p-value derived after correction at the voxel level at Z>2.3 and cluster level thresholding at p<0.025 (FWE).

***Corrected p: p-value after subsequent correction for familiality and medication use.

+Effects represent altered activity in the Reward Cue > Non-Reward Cue contrast.
