# Supplementary material for: Distinct effects of ASD and ADHD symptoms on reward anticipation in participants with ADHD, their unaffected siblings and healthy controls: a cross-sectional study
Source: Mol Autism. 2015 Aug 28;6:48. doi: 10.1186/s13229-015-0043-y (PMC4551566; doi:10.1186/s13229-015-0043-y)
Supplement: Additional file 5: — Table of MRI results. Includes additional information about the main MRI results of the current study. (PDF 230 kb) [file 13229_2015_43_MOESM5_ESM.pdf]

Additional File 5

Table of MRI results.

*MNI coordinates refer to the cluster maximum in MNI space
**Initial p: cluster p-value derived after correction at the voxel level at Z>2.3 and cluster level thresholding at p<0.025 (FWE)
***Corrected p: p-value after subsequent correction for familiality and medication use.
+Effects represent altered activity in the Reward Cue > Non-Reward Cue contrast
NA=Not applicable; (bil.)= bilateral.

**REWARD ANTICIPATION**					
Contrast	Cluster location	MNI coordinates* (X Y Z)	Cluster extent (voxels)	Initial p**	Corrected p***
Reward Cue > Non-Reward Cue	Brain-wide, including:<br>Occipital cortex (bil.)<br>Posterior parietal cortex (bil.)<br>Parahippocampal cortex (bil.)<br>Fusiform cortex (bil.)<br>Cerebellum<br>Sensorimotor cortex (bil.)<br>Posterior, mid and anterior cingulate cortex<br>Insular cortex (bil.)<br>Basal ganglia<br>Left midfrontal gyrus	14 -76 8	72250	3.83×10^{-28}	NA
Non-Reward Cue > Reward Cue	Left inf. frontal gyrus<br>Medial prefrontal gyrus	-40 40 -12	19571	1.56×10^{-11}	NA
	Posterior cingulate and retrosplenial cortex	2 -58 40	3560	0.002	NA
	Bilateral hippocampus	24 -12 -18	2747	0.008	NA
Positive effect of ASD symptoms+	Left insula	-44 -24 6	208	3.4×10^{-6}	0.002
Negative effect of ADHD symptoms+	Left dorsolateral prefrontal cortex	-34 22 30	161	7.65×10^{-5}	0.003

REWARD OUTCOME				
Contrast	Cluster location	MNI coordinates* (X Y Z)	Cluster extent (voxels)	Initial p**
Reward Hit-Miss > Non-reward Hit-Miss	Brain-wide, including:<br>Inferior parietal cortex (bil.)<br>Posterior parietal cortex (bil.)<br>Lateral occipital cortex (bil.)<br>Posterior, mid and anterior cingulate gyrus<br>Orbitofrontal cortex<br>Medial prefrontal cortex<br>Striatum<br>Amygdala (bil.)<br>Hippocampus (bil.)	-46 -56 -14	53407	3.95×10^{-29}
Non-reward Hit-Miss > Reward Hit-Miss	NA	NA	NA	NA
