# Supplementary material for: Distinct effects of ASD and ADHD symptoms on reward anticipation in participants with ADHD, their unaffected siblings and healthy controls: a cross-sectional study
Source: Mol Autism. 2015 Aug 28;6:48. doi: 10.1186/s13229-015-0043-y (PMC4551566; doi:10.1186/s13229-015-0043-y)

Additional File 6. Comparison of effects in Adults and Participants under 18

<u>ASD Symptoms</u>

Y-axes represent the left insular parameter estimates for each participant. X-axes represent their raw CSQBASD score. Effect of ASD symptoms in participants under 18 (n=179): p=0.008; Effect of ASD symptoms in adult participants (n=145): p=0.168. Effects are corrected for Age, Gender, IQ, Scansite, ADHD Symptoms, ODD/CD comorbidity, and Family ID. Explicitly including the factor Adults vs Children and its interaction with CSBQASD scores in the model above showed that there was no significant difference between the two age groups in the effect of ASD symptoms on insula activity during reward anticipation (p>0.05).

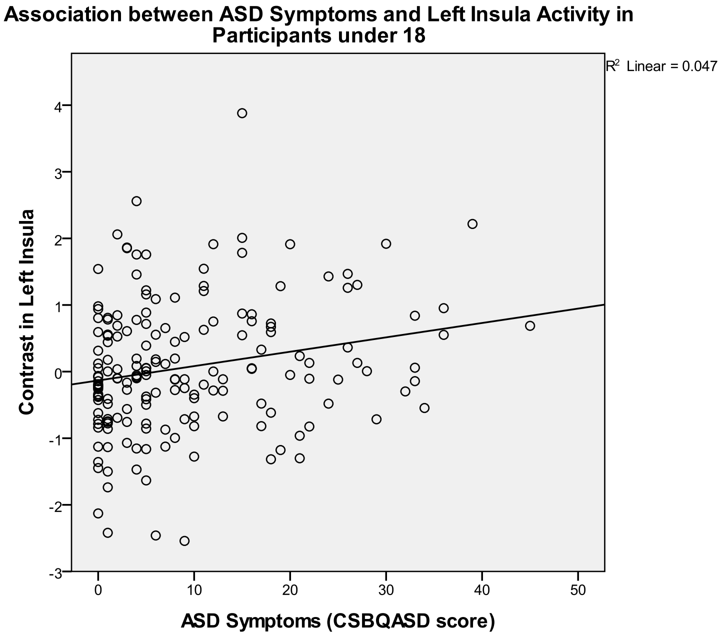

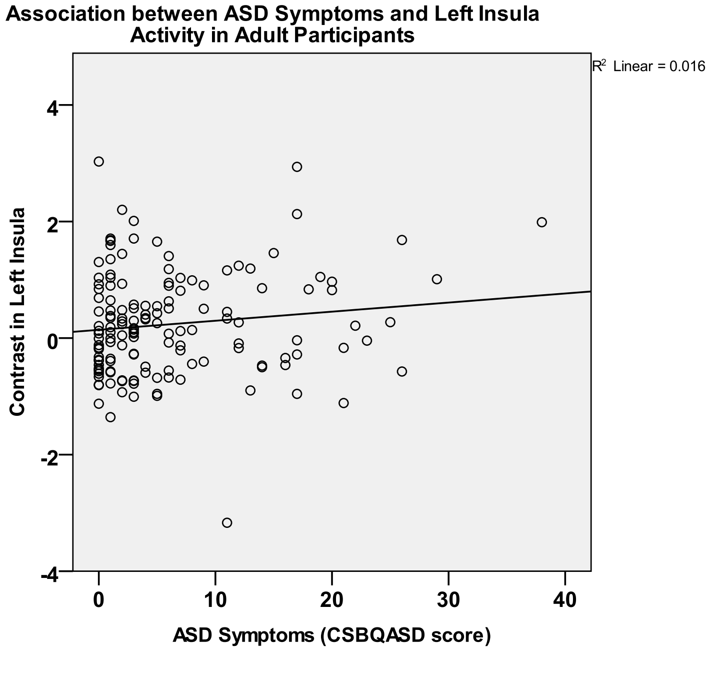

ADHD Symptoms

Y-axes represent the left dorsolateral prefrontal cortex parameter estimates for each participant. X-axes represent the sum of the raw CPRS-L scores on the inattentive and hyperactive/impulsivity subscales. Effect of ADHD symptoms in participants under 18 (n=179): p=0.039; Effect of ADHD symptoms in adult participants (n=145): p=0.025. Effects are corrected for Age, Gender, IQ, Scansite, ASD Symptoms, ODD/CD comorbidity and Family ID. Explicitly including the factor Adults vs Children and its interaction with ADHD Symptoms in the model above showed that there was no significant difference between the two age groups in the effect of ADHD symptoms on mid frontal activity during reward anticipation (p>0.05).

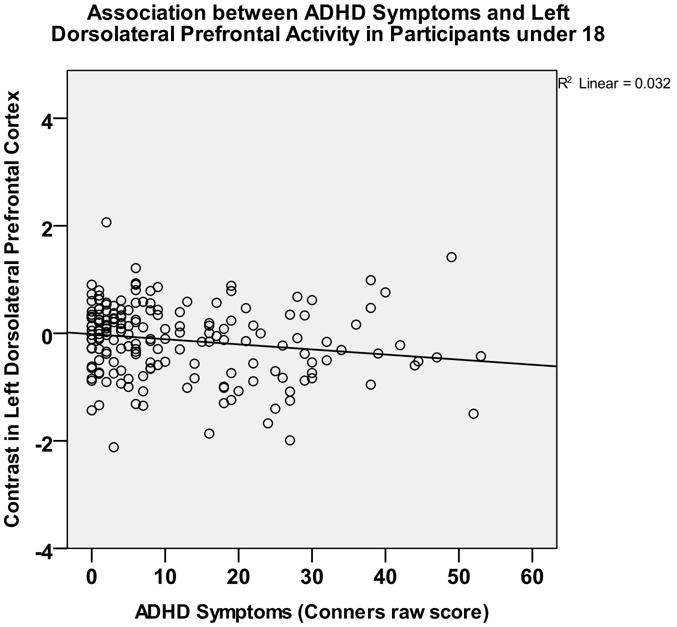

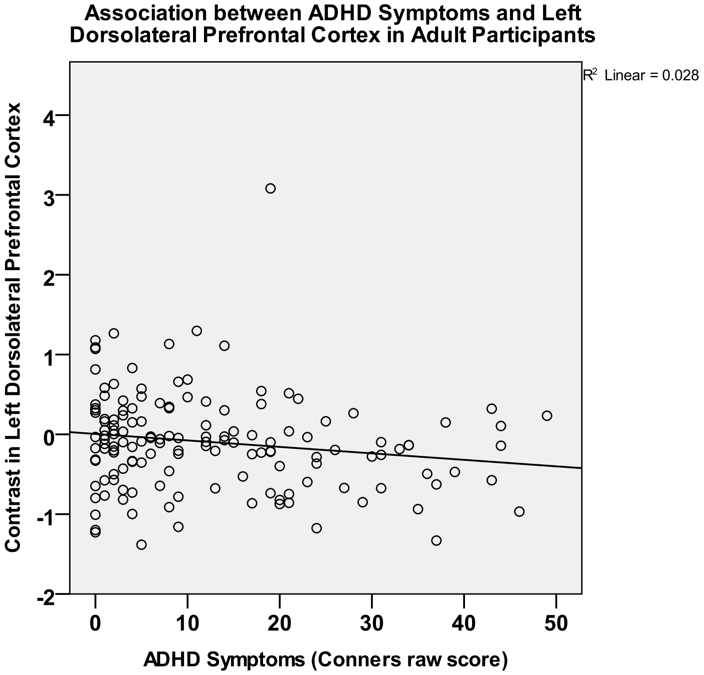

Supplement: Additional file 6: — Comparison of effects in Adults and Participants under 18. Includes additional information about and results from the comparison of ADHD and ASD effects in adults and participants under 18. (PDF 485 kb) [file 13229_2015_43_MOESM6_ESM.pdf]
