# Supplementary material for: Distinct effects of ASD and ADHD symptoms on reward anticipation in participants with ADHD, their unaffected siblings and healthy controls: a cross-sectional study
Source: Mol Autism. 2015 Aug 28;6:48. doi: 10.1186/s13229-015-0043-y (PMC4551566; doi:10.1186/s13229-015-0043-y)
Supplement: Additional file 7: — Plots of ASD and ADHD effects in the three experimental groups. Contains plots of the effects of ASD and ADHD on reward anticipation split across the three experimental groups. (PDF 317 kb) [file 13229_2015_43_MOESM7_ESM.pdf]

Additional File 7. Plots of ASD and ADHD effects in the three experimental groups

Effect of ASD Symptoms

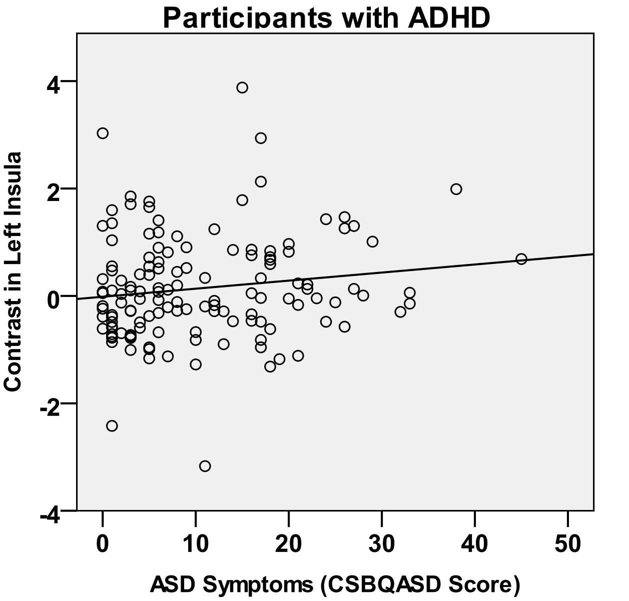

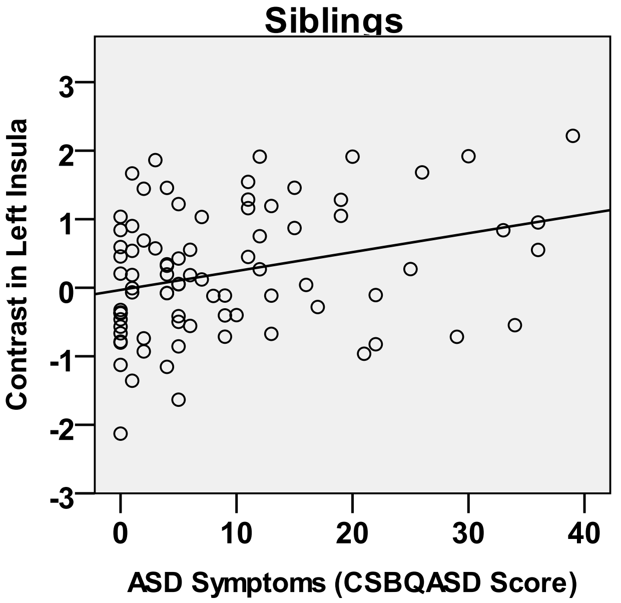

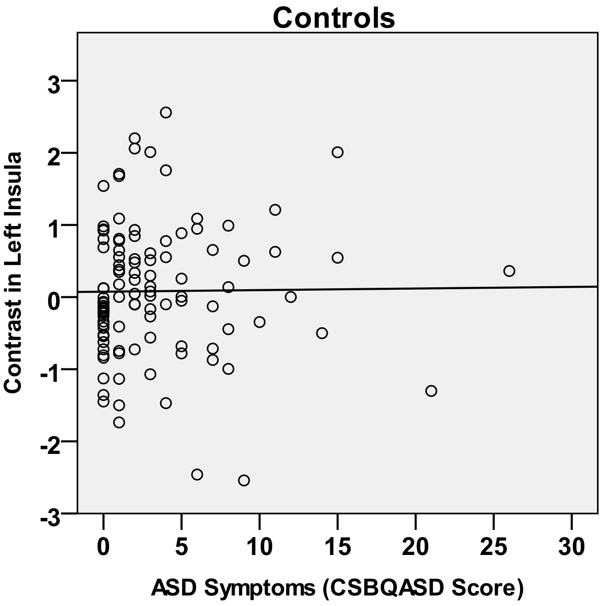

$\mathbf{R^2_{ADHD}=0.021}$

$\mathbf{R^2_{SIBLINGS}=0.092}$

$\mathbf{R^2_{CONTROLS}=0.001}$

Effect of ADHD Symptoms

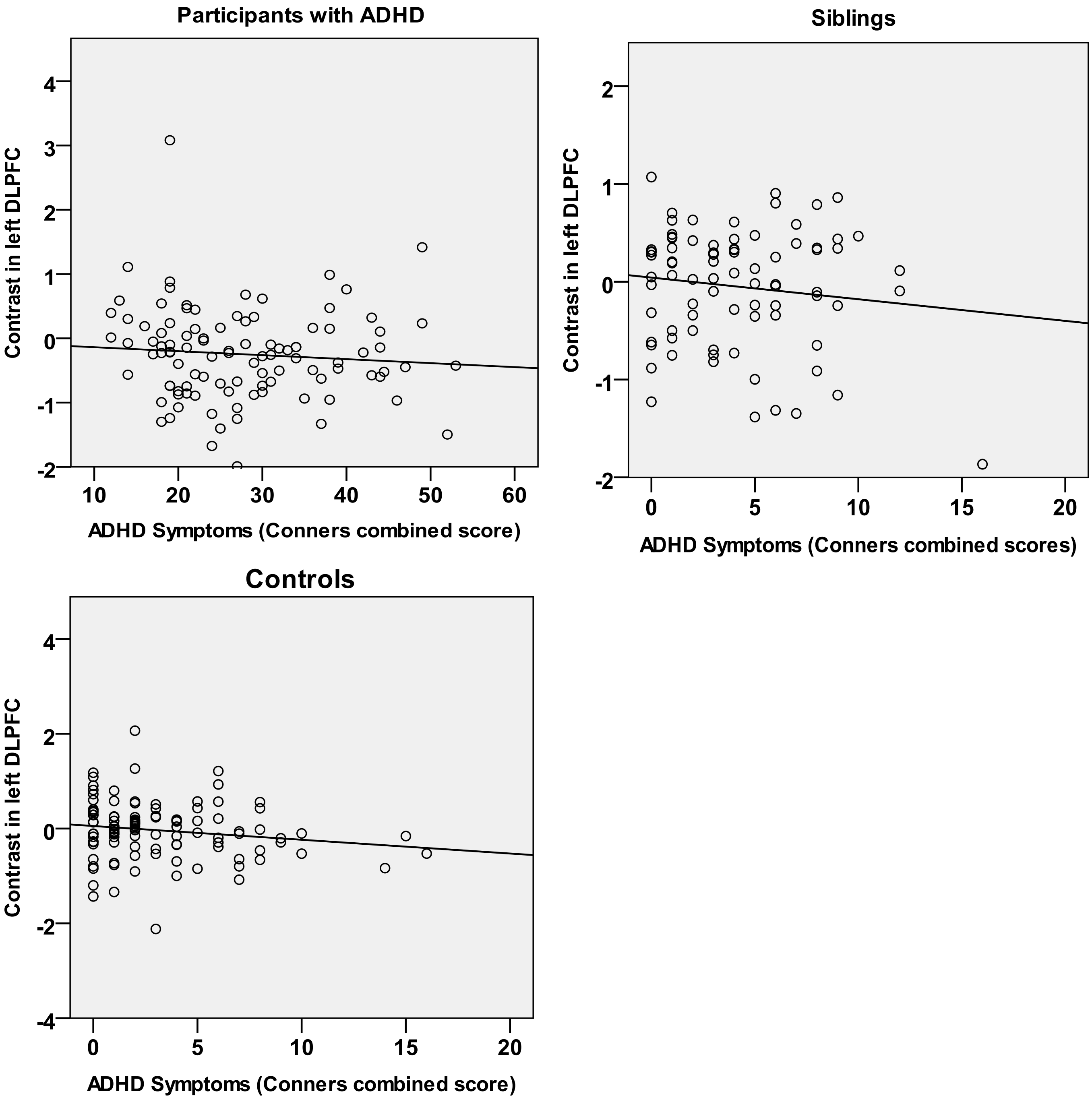

R^2_{ADHD}=0.007

$R^2_{SIBLINGS}$=0.016

$R^2_{CONTROLS}$=0.026
